# What can we learn from the Baduanjin rehabilitation as COVID‐19 treatment?: A narrative review

**DOI:** 10.1002/nop2.1572

**Published:** 2022-12-27

**Authors:** Zhenggang Zhu, Xiaoyan Pan, Faping Zhong, Jun Tian, Marilyn Li Yin Ong

**Affiliations:** ^1^ Exercise and Sports Science Programme, School of Health Sciences Universiti Sains Malaysia Kubang Kerian Kelantan Malaysia; ^2^ School of Nursing Hunan University of Chinese Medicine Changsha Hunan China; ^3^ Department of Respiratory Medicine The First Hospital Changde City of Traditional Chinese Medicine Changde Hunan China; ^4^ Department of Geriatric Medicine Xiangya Hospital of Central South University Changsha Hunan China; ^5^ School of Sport Exercise and Health Sciences Loughborough University Leicestershire UK

**Keywords:** Baduanjin, COVID‐19, exercise, rehabilitation

## Abstract

**Aim:**

To understand Baduanjin rehabilitation therapy in mild COVID‐19 patients.

**Design:**

A narrative review.

**Methods:**

A literature search for COVID‐19 and Baduanjin treatments was conducted on Chinese and English electronic databases: China National Knowledge Infrastructure, Wanfang Data, Embase, PubMed, Scopus, Science Direct, Ebscohost, SPORTDiscus and ProQuest.

**Results:**

Twelve studies on the Baduanjin rehabilitation for COVID‐19 patients have been included. We acknowledged the considerable published research and current clinical practice using Baduanjin for COVID‐19 treatment in the following areas: anxiety, depression, insomnia, lung function rehabilitation, immunity and activity endurance.

**Conclusion:**

The use of Baduanjin as adjuvant therapy for COVID‐19 patients' rehabilitation is still limited, therefore, more clinical studies are needed to confirm its efficacy.

## 1 BACKGROUND

Traditional Chinese medicine (TCM) played an important role in the Hubei Province during the COVID‐19 pandemic as it was included in the guideline for COVID‐19 diagnosis and treatment in China (Ren et al., 2020). TCM therapies, such as Chinese herbal decoction, massage, Guasha‐scraping, pressure point application, acupuncture, TCM nursing technology techniques and Baduanjin exercise therapy, were widely used in “fangcang” hospitals, which were set up to block the spread of infection and treat mild patients (Chen & Chen, 2020; Yang, Islam, et al., 2020; Yang, Luo, et al., 2020). In a quote, TCM research led by the top respiratory expert, Dr Zhong Nanshan, suggested that the best way to resist diseases was to exercise and improve immunity. Hence, he advocated practising Baduanjin, a medical sports and therapy treasure that originated 800 years ago in the Northern Song Dynasty (Tengxun news, 2020).

Baduanjin is a traditional Chinese low‐impact aerobic exercise therapy for enhancing physical and mental health and has been used to rehabilitate COVID‐19 patients as an effective and safe therapeutic method (Jing et al., 2018; Ma et al., 2020). This narrative review's objective was to explore the evidence of using Baduanjin exercise rehabilitation to improve the health outcomes of COVID‐19 patients.

## 2 THE HEALTH OUTCOMES DUE TO COVID‐19 ISOLATION AND RECOVERY

The sudden outbreak of COVID‐19 causes an emotional response of extreme fear and uncertainty in public could affect COVID‐19 patients directly or indirectly. Isolation practices may have led to negative social behaviour and mental health even after discharging from the hospital. In recent studies, “long COVID” has been coined to describe people who recovered from COVID‐19 but still have lasting symptoms after the clinical onset of the infection, such as anxiety, depression, sleep difficulties, fatigue and muscle weakness (Bellan et al., 2021; Huang et al., 2021).

A retrospective cohort study reported that the frequency of long COVID was 64.7%. The most common long COVID symptoms were fatigue (45.9%), respiratory distress (25.6%) and muscle/joint pain (24.8%), respectively (Yaksi et al., 2022). Furthermore, due to the enduring epidemic period, overworked healthcare workers may influence timely and effective treatments of COVID‐19 patients. The prevalence of depression, anxiety and stress were 258 (85.72%), 189 (62.80%) and 151 (49.84%), respectively, among nurses in COVID‐19 hospitals (Bhandari et al., 2022).

Considering that huge physical and psychological impact COVID‐19 has caused, there is an urgent need for a simple, reliable and feasible treatment to improve the physical and mental health of recovered COVID‐19 patients, including medical staff and family members. One such treatment that can often be performed by oneself at home with limited supervision is Baduanjin.

## 3 INTRODUCTION TO BADUANJIN

Baduanjin exercise has been described as a combination of Confucianism, Buddhism and Taoism, known as the “three pillars” of ancient Chinese society's philosophical and religious teachings and TCM (Li, 2015). Therefore, the influence of these teachings has produced a manner in which, when practising Baduanjin, the primary focus is self‐regulation. To sense the flow of Qi through the body, the practitioner must clear their mind of all thoughts and concentrate on a specific body location (Chen et al., 2006; Koh, 1982). Baduanjin consists of eight movements, each of which takes 2–3 min to complete, and the whole set of movements lasts about 20 min. During practice, it emphasizes firm yet gentle body and limb movements, guided by a calm mind and awareness of spontaneous breathing to cultivate the coordination between the mind and body (Ng & Tsang, 2009). The low‐intensity nature, relatively comfortable physical posture, and movements of Baduanjin are considered appropriate without fatiguing and aggravating the mild symptoms of COVID‐19 patients performing the exercise (Li et al., 2014). In addition, there were no statistically significant adverse events associated with practising Baduanjin in previous studies, such as falls or physical injuries, which suggested that Baduanjin exercise is safe for the public, especially for the elders, which make up most of the COVID‐19 patients (Fan et al., 2020).

The rationale for using Baduanjin to treat COVID‐19 symptoms, stems from the concept of Qi, which is involved in regulating the body's physiological and pathological mechanisms. Baduanjin rehabilitation is given as an adjuvant mind–body exercise therapy based on regulating Qi movement through the body channels known as the meridians. These meridians are distributed at the surface areas of the limbs and trunk, which are connected to the internal organs. Hence, Baduanjin exercise is geared towards meridian conditioning, which regulates the physical function and thus promotes disease recovery (Han et al., 2020). Given the impact of COVID‐19 on patients' quality of life, mental and physical health, meridian conditioning may improve mental and physical functioning. Baduanjin is seen as a low‐cost, convenient and conducive method to rehabilitate patients due to its appeal as a non‐invasive method with characteristics of good analgesic effects (Ma et al., 2020).

**FIGURE 1:**
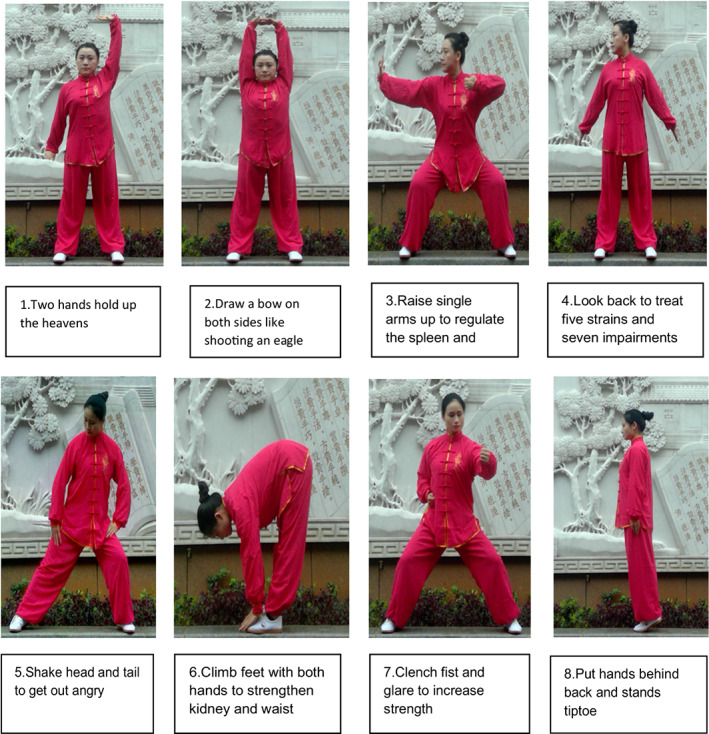
Eight fundamental movements of Baduanjin. Source courtesy of Duan Xiaocheng from the First Affiliated Hospital of Hunan University of Chinese Medicine, Hunan Province, China

Baduanjin, which translates to “eight pieces of brocade” or “eight silken movements,” has eight fundamental fluid movements. The first movement is the “two hands holding up the heavens,”, which according to TCM, this form can relieve fatigue and eliminate weariness and also helps to rebuild an erect posture of the chest and back. The second movement, called “drawing a bow on both sides like shooting an eagle,” is believed to enhance the functions of the respiratory and circulatory systems. The third movement, “raising single arms up” is said to regulate the spleen and stomach to strengthen the digestive system and the muscles in the arms and shoulders. The fourth movement, “looking back to treat five strains and seven impairments,” is said to help strengthen the neck muscles and stimulate the central nervous system, including preventing cervical‐vertebral fatigue. The fifth movement, “shaking head and tail to get out angry” relieves tension in the nervous system and adjusts the spine. The sixth movement, “climbing feet with both hands to strengthen kidney and waist,” stretches the waist and strengthens the kidney function. The seventh movement, “clenching fist and glare to increase strength,” is believed to improve muscular strength and endurance. The last movement, “putting hands behind the back and standing tiptoe,” helps strengthen the spine's ligaments (Qiu, 2009). The fundamental movements of Baduanjin are illustrated in Figure 1.

## 4 METHODS

### 4.1 Review design and literature search

This narrative review is based on the underpinning of the need for a non‐invasive and effective rehabilitative method. Baduanjin, which is currently applied in China since the outbreak of COVID‐19, was chosen for this review to explore the Baduanjin‐induced rehabilitative method and its effect on the clinical implications of COVID‐19 recovery. This review was also conceptualized as part of the World Health Organization (WHO) initiatives on traditional medicine strategy 2014–2023, focusing on the evidence and learning, data and analytics, sustainability and equity, as well as innovation and technology to optimize the contribution of traditional medicine to global health and sustainable development, while respecting the local heritages, resources and rights as a guiding principle (World Health Organization, 2013). A systematic and comprehensive search of Chinese and English electronic databases was conducted: China National Knowledge Infrastructure (CNKI), Wanfang Data, PubMed, Scopus, Science Direct, Ebscohost, SPORTDiscus and ProQuest. Articles published from December 2019 to August 2022 were retrieved. The key search terms used for searching the articles were (COVID‐19 OR coronavirus disease 2019 OR SARS‐CoV‐2 Virus) and (Baduanjin) and (patient*). Two researchers (ZZ and JT) independently searched for the articles.

### 4.2 Inclusion and exclusion criteria

Only original studies such as randomized controlled trials (RCT), pre and post‐comparisons, cohort studies and retrospective analyses were included in this review. Other studies such as meta‐analyses, meta‐syntheses, scoping reviews, narrative reviews, rapid reviews, critical reviews, integrative reviews, guidelines and protocols were excluded. Citations retrieved from the initial database search were excluded if the publications were abstracts, conference proceedings, editorials, letters to editors, research letters or short communication and opinion articles.

### 4.3 Data extraction

**FIGURE 2:**
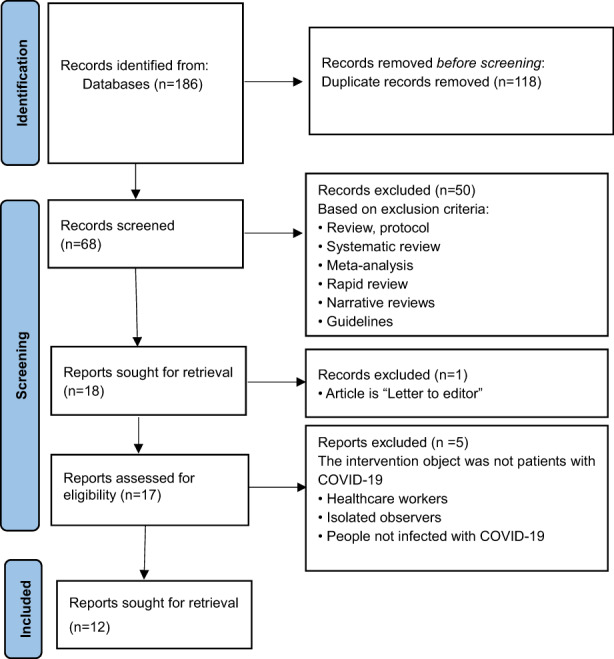
Search strategy development and findings

Key items from the included articles, including author (s), year of publication, study design, the number of participants (sample), setting, interventions and main outcomes, were extracted by ZZ. The articles and items were appraised by two investigators (MLYO and XP) based on the inclusion and exclusion criteria. Any disagreements were resolved by consensus in the presence of a third investigator (FZ). The team then critically discussed and approved the data from all the included studies. The process of the literature search and findings is given in Figure 2. The key information is summarized in Table 1.

**TABLE 1:** Summary of Baduanjin intervention on the main outcomes in COVID‐19 patients

Author and publication year	Study design and implementation date	Clinical setting	Sample	Style	Intervention	Main outcomes
Chen, Yu, et al. (2020)	Randomized controlled trial February to March 2020	Guangdong Provincial Hospital of Traditional Chinese Medicine	*n* = 59 M/F = 24/35 Age range 33–77 yo IG: 61.40 ± 16.0 yo CG: 60.10 ± 15.1 yo	Baduanjin	IG: Routine symptomatic treatment, Baduanjin was practiced twice a day, once in the morning and once in the evening, 15–30 min each time, for a total of 7 days, in addition to routine symptomatic treatment. CG: Routine symptomatic treatment and usual nursing care	Baduanjin improved fatigue and anxiety symptoms in patients with COVID‐19
Chen, Chen, and Pan	Randomized controlled trial February to March 2020	Wuhan *Jinyintan* Hospital	*n* = 29 M/F = 13/16 Age range 39–74 yo IG: 68.5 ± 10.80 yo CG: 67.6 ± 11.2 yo	Baduanjin	IG: Adapted Baduanjin in bed was practiced 20–30 min, twice a day, for a total of 21 days, in addition to routine symptomatic treatment. CG: Routine symptomatic treatment	Adapted Baduanjin in bed improved anxiety and depression
Peng et al. (2021)	Randomized controlled trial February to March 2020	Wuhan *Fangcang* hospital	*n* = 65 M/F = 26/39 Age range 18–88 yo IG: 61.38 ± 2.83 yo CG: 54.57.6 ± 19.80 yo	Baduanjin combined *Zusanli* acupoint massage	IG: The third section movement of Baduanjin and Zusanli three times a day, 3 min each time, for 7 days. CG: Conventional therapy	Baduanjin combined with *Zusanli* acupoint massage improved appetite and promoted digestion
Wan (2020)	Randomized controlled trial February 2020	Affiliated Hospital of Jiangxi University of Traditional Chinese Medicine	*n* = 16 Age range: 30–68 yo IG: 39.86 ± 3.62 yo CG: 40.17 ± 3.19 yo	Baduanjin	IG: Baduanjin was practiced 3 times a day for 7 days in addition to routine symptomatic treatment. CG: Routine symptomatic treatment	Baduanjin relieved dyspnoea and promoted recovery
Wang et al. (2021)	Randomized controlled trial February to March 2020	Wuhan *Fangcang* hospital	*n* = 60 M/F = 32/28 Age range 18–75 yo IG: 63.41 ± 6.73 yo CG: 63.42 ± 6.91 yo	Baduanjin combined five‐element music	IG: Baduanjin was practiced twice a day for 30 min each time, followed by listening to five‐element music 30 min, twice a day for a total of 14 days. The volume of music was below 50 dB. CG: Routine treatment and care	Baduanjin combined with five‐element music reduced anxiety and depression while improving sleep quality
Wu, Pan, et al. (2020); Wu, Shan, & Tang (2020)	Randomized controlled trial February 2020	Wuhan *Fangcang* hospital	*n* = 120 Mean age: 51.30 ± 8.45 yo	Baduanjin	IG: Baduanjin was practiced 30–60 min, twice a day for a total of 15 days, in addition to routine symptomatic treatment. CG: Symptomatic treatment and usual daily activities	Baduanjin relieved anxiety symptom and promoted the early recovery of patients in COVID‐19 shelter hospitals
Xu (2020)	Pre‐post comparison February to March 2020	Wuhan *Fangcang* hospital	*n* = 80 M/F = 38/42 Age range 27–65 yo Mean age: 41.05 yo	Baduanjin	IG: Baduanjin was practiced 40 min, once a day for a total of 7 days, in addition to routine symptomatic treatment. CG: Routine symptomatic treatment	Baduanjin improved the anxiety and depression of COVID‐19 patients isolated in shelter hospitals
(Yang, Islam, et al., 2020; Yang, Luo, et al., 2020)	Randomized controlled trial January to March 2020	Wuhan Red Cross Hospital	*n* = 110 M/F = 67/43 Age range 47–76 yo IG: 58.69 ± 5.41 yo CG: 58.23 ± 5.36 yo	Baduanjin combined with acupuncture	IG: Baduanjin was practiced 20 min a day for 30 days. Acupuncture on the *Dazhi* point and *Tiantu* point for 30 min, once a day for 30 days, in addition to routine symptomatic treatment. CG: Routine symptomatic nursing	Baduanjin combined with acupuncture improves patients' quality of life, reducing anxiety and depression
Yang et al. (2021)	Randomized controlled trial January to March 2020	Wuhan First Hospital	*n* = 87 M/F = 43/44 Age range 21–65 yo IG: 45 ± 11 yo CG: 45 ± 11 yo	Baduanjin and Auricular point compression	IG: Baduanjin was practised 20 min a day for 12 days, followed by 30 s auricular ear points compression with seeds, 3 times a day for 12 days, in addition to routine symptomatic treatment. CG: Oral estazolam 1 mg tablets given before bedtime to treat insomnia, once a day, for 12 days	Baduanjin combined with auricular point massage improved the sleep quality, anxiety and depression, immunity and insomnia of COVID‐19 patients. The curative effect was better than estazolam
Ye et al. (2020)	Retrospective analysis February to March 2020	Wuhan *Fangcang* hospital	*n* = 113 M/F = 1/112 Age range 20–77 yo Mean age: 46.66 ± 12.91 yo	Baduanjin	Baduanjin was practiced as a symptomatic treatment once a day for 1 h each time until discharged from the hospital(7~28 days)	Baduanjin was clinically effective for mild COVID‐19, reducing fatigue and shortness of breaths after physical activities, preventing exacerbations and relieving patients' psychological stress
Yin et al. (2020)	Randomized controlled trial February to March 2020	Xiaogan First People's Hospital	*n* = 40 M/F = 21/19 Age range: 20–66 yo IG: 47.10 ± 10.99 yo CG: 43.95 ± 13.75 yo	Baduanjin and five‐element music	IG: The observation group was given five‐element music therapy and Baduanjin. Baduanjin was practiced 60 min per day, 5 times a week for a total of 2 weeks. Five‐element music therapy was played from 17:00 to 18:00 every day, once a day, 30 min each time, for a total of 2 weeks, in addition, to routine symptomatic treatment. CG: Routine symptomatic treatment and the usual daily lifestyle were maintained	Combined with five‐element music, Baduanjin alleviated negative emotions such as anxiety and depression and improved the quality of life in COVID‐19 patients
Zhang et al. (2021)	Self before and after comparison February to March 2020	Wuhan *Fangcang* hospital	*n* = 91 M/F = 40/51 Age range:20–77 yo	Baduanjin	Baduanjin exercise was done 20 min, 4 times a day for a total of 18 days	Baduanjin alleviated the anxiety and depression symptoms of the patients in the shelter hospital

*Note*: Age is presented as mean ± standard deviation.

Abbreviations: CG, control group; F, female; IG, intervention group; M, male; yo, years old.

## 5 RESULTS

Twelve articles were identified and included in this review's final stage of the screening procedure. One paper was published in English, while the other articles were published in Chinese. All the studies were implemented between January and March 2020, which is during the peak period of COVID‐19 outbreak in Wuhan, China. Six of the studies were conducted in the Wuhan *Fangcang* hospital, a temporary hospital built for isolation and treatment of COVID‐19.

Most of this research was randomized, controlled trial studies (9 studies, 75%), while two were pre‐post comparison studies (16.6%), and one was retrospective analysis (8.4%). The number of participants in the studies ranged from 16 to 120. In this review, the total number of participants was 810. The average sample size of each study was 68.

Twelve studies were included, seven of which only used Baduanjin, and the remaining five combined Baduanjin with other treatments (two are Baduanjin combined with five‐element music, the other two are Baduanjin combined with acupoint massage, one is Baduanjin combined with auricular point massage). The duration of the programs ranged from seven to 30 days, and the frequency was between one and four times a day, 5–7 days a week. Each exercise lasted between 20 to 60 min.

From the main findings in Table 1, we found that the main efficacy of Baduanjin on patients with COVID‐19 is reflected in four aspects: first, to improve anxiety and depression; second, to relieve the symptoms of dyspnoea. The third is to improve the symptoms of insomnia and sleep quality; the fourth is to strengthen immunity; the fourth is to enhance activity endurance.

## 6 DISCUSSION

### 6.1 Baduanjin for relieving symptoms of COVID‐19 related anxiety and depression

Baduanjin is a COVID‐19 rehabilitation exercise method recommended by the Chinese Rehabilitation Society (Wu, 2020a, 2020b). It has also been demonstrated to reduce depression and anxiety symptoms in patients with psychosomatic illnesses (Xu, 2020; Zhang et al., 2021), which may be appropriate as an alternative therapy for rehabilitation in COVID‐19 patients with these symptoms (Ma et al., 2020).

During the outbreak of COVID‐19, the nationwide lockdown imposed on the public led to a series of psychosocial problems, such as self‐harm, domestic violence or aggression due to lockdown, grieving of family and friends who were infected or died due to COVID‐19, separation from family and friends and social isolation (Choi et al., 2020; Taylor, 2019). These conditions led to a higher incidence of anxiety and depression than before the COVID‐19 pandemic. One investigation reported that 402 adults surviving COVID‐19 had 31% depression and 42% anxiety in Italy (Mazza et al., 2020). Preliminary data suggests that 19% of citizens had depression and 14% had anxiety in Hong Kong during the pandemic (Choi et al., 2020). Adverse emotions such as anxiety and depression have negatively affected the rehabilitation of patients. One study reported that Baduanjin could alleviate the anxiety symptoms of perimenopausal women more than walking exercises (Xiao et al., 2016). Another study identified that Baduanjin is a better method of exercise than jogging in delaying cognitive and physiological function decline in the elderly (Sun & Wang, 2008). Baduanjin has shown a statistically significant effect in improving patients' negative emotions. Xu (2020) described that the Baduanjin training intervention improved patients' ability to focus attention while in isolation at the shelter hospital. In addition, the author stated that the patient's mental status improved, relieving their anxiety and depression and thus, increasing their confidence to overcome the disease. Performing regular Baduanjin exercise for at least 60 min per day, three times per week (720 MET‐min per week) for 8 weeks showed a positive trend towards improving self‐image and thus, reducing psychologic distress compared to the inactive controls (Lee et al., 2021; Wang, 2011). Research has shown that a MET‐min/week range of 500 to 1000 is the most beneficial to COVID‐19 patients (Lee et al., 2021). These positive effects can be attained when coordination between breathing, body movement and mind is achieved harmoniously after several Baduanjin practice sessions (Yang & Wei, 2012). There are also fewer cognitive demands when practicing Baduanjin. Therefore, it is considered beneficial to the mental health of COVID‐19 patients (Han et al., 2020).

Compared to other aerobic exercises, Baduanjin exercise emphasizes slow and deep breathing, a calm mind and circulation of *Qi* in the body. Baduanjin exercise can coordinate the mind and body by controlling the flow of *Qi* in the body (Liu et al., 2014; Qi et al., 2018). It focuses on the mobilization of functional potentialities, making the process of breathing smoother and unifying the mind and body by regulating breathing, thereby promoting the normal circulation of *Qi* (Zheng et al., 2015). The second movement of Baduanjin is “like shooting from left to right,” which helps expand the thorax and mobilize the diaphragm. Subsequently, the range of motion is significantly increased, the diaphragm function is improved, and the vital capacity increases (Qi et al., 2018). Participants felt their bodies relaxed, moods calm, and passive emotions improved after regular Baduanjin training for several weeks (Yang, Islam, et al., 2020; Yang, Luo, et al., 2020).

### 6.2 Baduanjin for easing COVID‐19 related dyspnoea and wheezing symptoms

The chest imaging of moderate‐to‐severe COVID‐19 patients showed multiple fibrous streaks and infiltrating lesions, which may progress to impaired lung function leading to dyspnoea and asthenia (Zhu et al., 2020). However, radiographic imaging is insensitive to early or mild COVID‐19 symptomatic infections, although the disease can be detected and monitored for abnormality or progression using computed tomography (CT) (Rubin et al., 2020). For mild patients, practicing Baduanjin exercise has a positive effect in alleviating the symptoms of dyspnoea and wheezing during the rehabilitation period (Zhang, He, & Li, 2020; Zhang, Lu, et al., 2020; Zhao et al., 2020). Cai et al. (2021) identified Baduanjin as an adjuvant treatment for COVID‐19 to significantly improve the patient's shortness of breath symptoms. A study showed that pulmonary function improved after 3 months of Baduanjin intervention in the elderly Zhou et al., (2016) and Chen, Yu, et al. (2020) suggested that Baduanjin exercise could improve the fatigue state of COVID‐19 patients and promote recovery. The improved recovery may be attributed to the practice of these Baduanjin exercises: “two hands hold up the heavens,” “separate heaven and earth,” and “two hands hold the feet to strengthen the kidneys and waist,” in which the respiratory rate and depth may be enhanced through the increase in the diaphragm movement (Jing et al., 2018). By performing these particular exercises, the respiratory muscles are trained, thereby increasing lung capacity. Taken together, the combination of breathing movements of the chest and abdominal wall increases the activity of the diaphragm and abdominal muscles, thereby regulating the intercostal muscles and respiratory function, causing a further increase in pulmonary ventilation (Deng & Chen, 2015; Han & Lin, 2016; Zhang, 2012).

### 6.3 Baduanjin for improving sleep quality in COVID‐19 patients

Insomnia is the most notable mental health condition among the affected COVID‐19 population, showing a 23.87% prevalence with reports of significantly higher insomnia among healthcare workers than in the general population (Cenat et al., 2020). Killgore et al. (2020) reported that people are experiencing more sleep‐related problems due to pandemic‐related anxieties in the United States. Additionally, statistical analysis revealed that insomnia severity was greater when associated with pandemic fears and suicidal thinking. Therefore, interventions aimed at improving sleep may be useful in reducing suicide risk during the current pandemic. Yang et al. (2021) described how COVID‐19 causes people to feel anxious and uneasy, even causing or aggravating insomnia, particularly in middle‐aged and older adults. In China, reports of insomnia in people aged 50 years or over account for 40% of total insomnia, whereas in the 60–90‐year‐old, the chronic insomnia rate was 90% (Li, 2020). Sleep deprivation will lead to a decline in natural killer (NK) cell activity and immune function. The NK cells are mainly involved in antiviral processes in vivo (Yang & Yu, 2017; Zhang et al., 2016). Therefore, COVID‐19 patients with immunodeficiency will experience an increase in dyspnoea and wheezing symptoms, complicating the medical prognosis and subsequently causing a higher risk of death (Zhang, Lu, et al., 2020).

Baduanjin can improve sleep disorders by regulating negative emotions, priming immune function and regulating various neurotransmitters (China Association of Acupuncture‐Moxibustion, 2020). Baduanjin exercise is a feasible, safe and effective intervention program for improving sleep quality in community‐dwelling older adults with sleep disturbances (Fan et al., 2020). Baduanjin's effect in promoting sleep quality may be due to enhanced levels of serum melatonin, an important hormone for regulating sleep–wake rhythm and modulating emotions (Simko et al., 2016). One research reported that Baduanjin exercise combined with ear acupoint stimulation could improve the sleep quality of COVID‐19 patients with insomnia, and the effect is better than the oral administration of estazolam (Yang et al., 2021). Estazolam medication possesses hypnotic and anti‐anxiety effects, but long‐term use can cause the suppression of respiration and drug dependence (Zhu & Yin, 2016). Hence, the non‐invasive nature of acupoint or pressure point (non‐needle) stimulation of specific body areas has been one of the many fundamental treatments of TCM, which complements seamlessly with Baduanjin exercise therapy (Lu et al., 2019).

### 6.4 Baduanjin for improving the immunity of COVID‐19 patients

**FIGURE 3:**
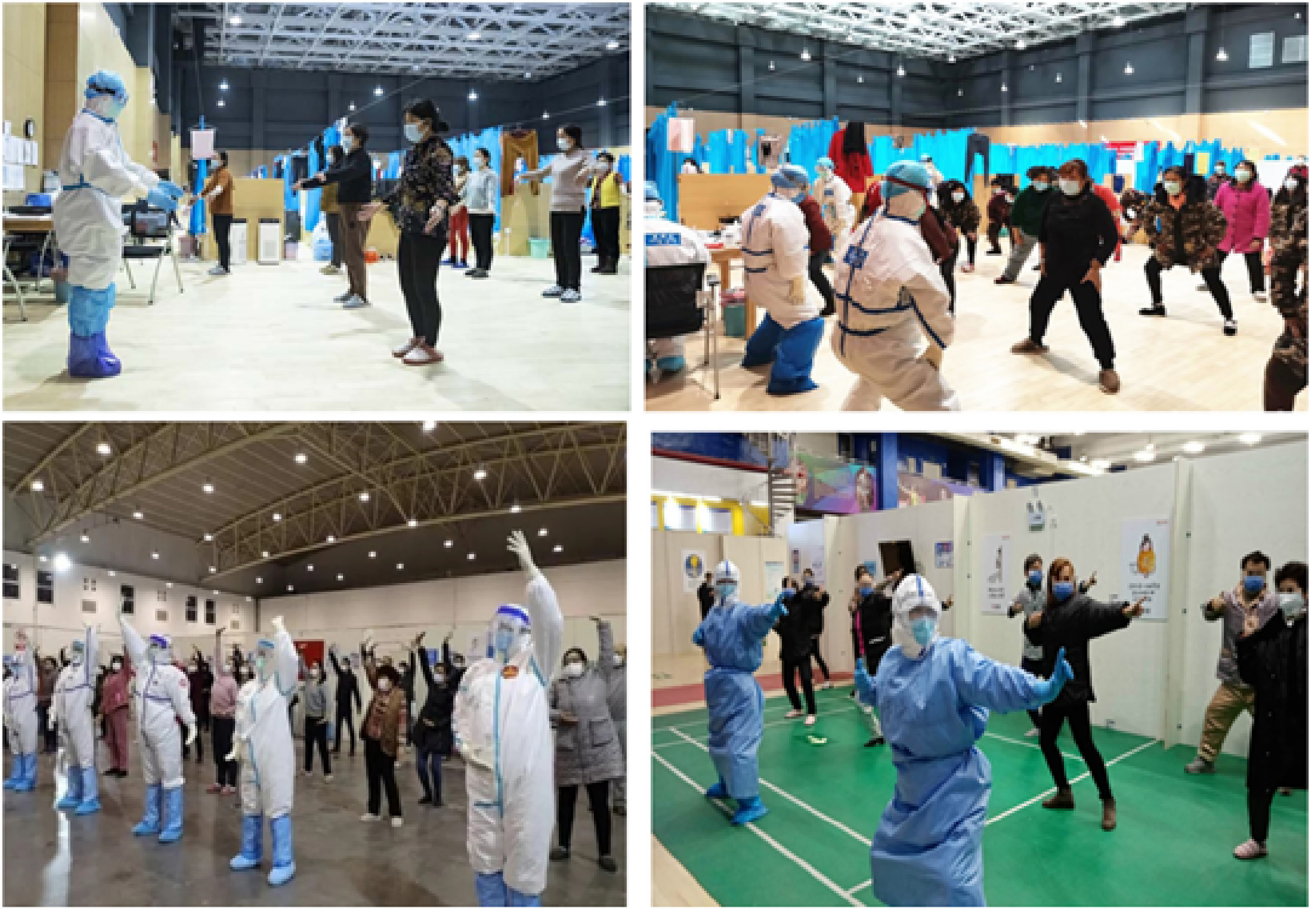
Medical staff taught Baduanjin to the COVID‐19 patients in Jiangxiao fangcang shelter hospital located in Hebei Province, China. Photo courtesy of Yinqin, a frontline nurse, working in the COVID‐19 shelter hospital in Wuhan

The immune system is an important defence against diseases. Immunity in western medicine theory is known as “Positive *Qi*” in traditional Chinese medicine theory (Wang & Hao, 2020). According to the ancient traditional Chinese internal medicine book Huangdi's Internal Classic or “Huangdi Neijing”, written during the period 475–225 BC, it was said that “If *Qi* is stored in the body, illness cannot occur” (Li, 2015). When the viscera function of the body is normal, the healthy *Qi* is sufficient, providing a solid and dense external body's immune defence system (first line of defence), thus the concept of “epidemic poison and evil *Qi*” is said to be presented with a difficult path to invade, and subsequently, the disease will not occur (Yao et al., 2020). Guided by the basic theory of TCM on the joint exercise of the essence, *Qi*, Baduanjin dredges the human body's meridians by stimulating the human body's healthy *Qi* and thus improves immunity and promotes the recovery of the body. Regular exercise can regulate the mental state and enhance the levels of T cells such as CD3+ (T helper and cytotoxic activator), CD4+ (T helper/regulatory), CD8+ (cytotoxic) and other immune cells in the patient's peripheral blood (Liang, 2018). In addition, college students practising 16 weeks of Baduanjin showed a statistically significant increase in serum immunoglobulin, IgM, the antibody produced by the adaptive humoral immunity in response to infection (Guo et al., 2020). These results provide strong evidence for Baduanjin to improve human immunity. Cao (2020) described how doctors and nurses led the COVID‐19 patients in Wuhan shelter hospital to practice Baduanjin as rehabilitation to promote recovery (Figure 3).

### 6.5 Baduanjin for improving the physical fitness of COVID‐19 patients

Mild COVID‐19 patients who are isolated without any form of physical rehabilitation may lead to poor physical fitness. Baduanjin exercise can be practised anywhere, even in a limited space such as the shelter hospital, hence it is suitable for COVID‐19 patients during the rehabilitation period. Baduanjin, which combines the “Qigong” theory (work energy) and aerobic exercise, is used as an integrative and complementary therapy in TCM to improve the pulmonary function of COVID‐19 patients (Ming, 2017; Wang & Meng, 2016) significantly. A meta‐analysis showed that Baduanjin could effectively improve patients' lung function and exercise tolerance or physical fitness, preventing muscle atrophy and inactivity (Cao et al., 2020). COVID‐19 patients' activity tolerance is closely related to lung function. Previous studies have shown that Baduanjin exercise helps improve pulmonary function, reduce fatigue and improve activity tolerance in mild or convalescent COVID‐19 patients (Bi & Guo, 2020; Chen, Yu, et al., 2020; Chen, Zhang, et al., 2020; Wu, Pan, et al., 2020; Wu, Shan, & Tang, 2020). Long‐term Baduanjin practice has been noted to enhance cardiac contractility, vascular filling, myocardial fibres and thus improve systemic circulatory function in the patients, thus may enhance the cardiovascular fitness of COVID‐19 patients (Zhang, 2012).

### 6.6 Limitation

This review has some limitations. Although most of the studies selected in this review were randomized and controlled trials, participants were not blinded and had to be conducted in shelter hospitals in isolation. In addition, the number of included studies was small, with only 12 studies eligible for inclusion, and there was a lack of large samples and high‐quality studies during the pandemic's restrictive movement orders. Third, there was heterogeneity about the duration of each exercise session and the number of exercises per day, which varied between studies. Fourth, some studies combined Baduanjin with other treatments, which cannot ascertain the Baduanjin effect alone, and these combined factors may weaken the strength of the evidence. Hence, the findings from this review may not be transferable to non‐clinical settings. However, with the gradual opening of the borders and the loosening of restrictions, more future studies can be conducted outside the confinement of the hospital and China. This review provides important implications for future COVID‐19 nursing practice as there is an opportunity for evidenced research to be conducted to ensure an enhanced recovery.

## 7 CONCLUSION

This review mainly introduces the important role of Baduanjin in rehabilitating mild COVID‐19 patients in China. Possible effects of Baduanjin include relieving symptoms of anxiety and depression, easing symptoms of dyspnoea and wheezing, improving sleep quality and enhancing activity endurance of mild COVID‐19 patients. The positive effects on the recovery of mild COVID‐19 patients reported in these limited Baduanjin studies warrant further analysis of larger data and clinical trials. Although Baduanjin may be prescribed internationally as an adjunct treatment for the rehabilitation of COVID‐19 patients, further clinical studies will be needed to understand Baduanjin's efficacy on different stages of COVID‐19 recovery, including research on socio‐medical aspects such as cultural acceptance and health policies.

## AUTHOR CONTRIBUTIONS

ZZ conceived the idea, gathered data, provided intellectual content and write the manuscript; XP provided intellectual content and support, appraised articles, wrote the manuscript. JT gathered the data, provided idea and reviewed the manuscript. MLYO provided idea, consultation and intellectual content, appraised articles, and wrote the manuscript; FZ provided idea, resolved disagreements and reviewed the manuscript. All authors approved the final version of the manuscript.

## FUNDING INFORMATION

This review is financially supported by the Novel Coronavirus Pneumonia Project, Hunan Provincial Science and Technology Department of the Chinese Government (NO.2020SK3033) and (NO.2020SK3050).

## CONFLICT OF INTEREST

There are no other conflicts of interest.

## ETHICAL STATEMENT

This review does not require ethical approval.

## Data Availability

The data that support the findings of this study are available from the corresponding author upon reasonable request.
